# Early postnatal care uptake and its associated factors following childbirth in East Africa—a Bayesian hierarchical modeling approach

**DOI:** 10.3389/fpubh.2024.1439280

**Published:** 2024-11-27

**Authors:** Bewuketu Terefe, Dejen Kahsay Asgedom, Fetlework Gubena Arage, Setognal Birara Aychiluhm, Tadesse Awoke Ayele

**Affiliations:** ^1^Department of Community Health Nursing, School of Nursing, College of Medicine and Health Sciences, University of Gondar, Gondar, Ethiopia; ^2^Department of Public Health, College of Medicine and Health Sciences, Samara University, Samara, Ethiopia; ^3^Department of Epidemiology and Biostatistics, Institute of Public Health, College of Medicine and Health Sciences, University of Gondar, Gondar, Ethiopia

**Keywords:** Bayesian hierarchical model, East Africa, factors, newborns, postnatal care, women

## Abstract

**Background:**

The postnatal period is a critical period for both mothers and their newborns for their health. Lack of early postnatal care (PNC) services during a 2-day period is a life-threatening situation for both the mother and the babies. However, no data have been examined for PNCs in East Africa. Hence, using the more flexible Bayesian multilevel modeling approach, this study aims to investigate the pooled prevalence and potential factors for PNC utilization among women after delivery in East African countries.

**Methods:**

We retrieved secondary data from the Kids Record (KR) demographic and health surveys (DHS) data from 2015 to 2022 from 10 East African countries. A total of 77,052 weighted women were included in the study. We used R 4.3.2 software for analysis. We fitted Bayesian multilevel logistic regression models. Techniques such as Rhat, effective sample size, density, time series, autocorrelation plots, widely applicable information criterion (WAIC), deviance information criterion (DIC), and Markov Chain Monte-Carlo (MCMC) simulation were used to estimate the model parameters using Hamiltonian Monte-Carlo (HMC) and its extensions, No-U-Turn Sampler (NUTS) techniques. An adjusted odds ratio (AOR) with a 95% credible interval (CrI) in the multivariable model to select variables that have a significant association with PNC was used.

**Results:**

The overall pooled prevalence of PNC within 48 hrs. of delivery was about 52% (95% CrI: 39, 66). A higher rate of PNC usage was observed among women aged 25–34 years (AOR = 1.21; 95% CrI: 1.15, 1.27) and 35–49-years (AOR = 1.61; 95% CrI: 1.5, 1.72) as compared to women aged 15–24 years; similarly, women who had achieved primary education (AOR = 1.96; 95% CrI: 1.88, 2.05) and secondary/higher education (AOR = 3.19; 95% CrI: 3.03, 3.36) as compared to uneducated women; divorced or widowed women (AOR = 0.83; 95% CrI: 0.77, 0.89); women who had currently working status (AOR = 0.9; 95% CrI: 0.87, 0.93); poorer women (AOR = 0.88; 95% CrI: 0.84, 0.92), middle-class women (AOR = 0.83; 95% CrI: 0.79, 0.87), richer women (AOR = 0.77; 95% CrI: 0.73, 0.81), and richest women (AOR = 0.59; 95% CrI: 0.55, 0.63) as compared to the poorest women; women who had media exposure (AOR = 1.32; 95% CrI: 1.27, 1.36), were having 3–5 children (AOR = 0.89; 95% CrI: 0.84, 0.94), had >5 children (AOR = 0.69; 95% CrI: 0.64, 0.75), had first birth at age < 20 years (AOR = 0.82; 95% CrI: 0.79, 0.84), had at least one ANC visit (AOR = 1.93; 95% CrI: 1.8, 2.08), delivered at health facilities (AOR = 2.57; 95% CrI: 2.46, 2.68), had average birth size (AOR = 0.94; 95% CrI: 0.91, 0.98) and small birth size child (AOR = 0.88; 95% CrI: 0.84, 0.92), had twin newborns (AOR = 1.15; 95% CrI: 1.02, 1.3), and fourth and above birth order (AOR = 0.88; 95% CrI: 0.82, 0.95) were individual-driven women who have been independently associated with PNC, respectively. Regarding community-level variables, rural women (AOR = 0.76; 95% CrI: 0.72, 0.79), high media exposure communities (AOR = 1.1; 95% CrI: 1.04, 1.18), communities with high wealth levels (AOR = 0.88 95% CrI: 0.83, 0.94), communities with high antenatal care (ANC) utilization (AOR = 1.13, 95% CrI: 1.07, 1.19), and long distance to health facilities (AOR = 1.5; 95% CrI: 1.38, 1.63) were among the community factors associated with PNC, respectively.

**Conclusion:**

One of the significant public health priorities in East Africa continues to be the underutilization of immediate PNC. The government ought to prioritize improving maternity and child health services, collaborating with interested parties in the area, reducing health disparities, educating mothers about child health, and other connected issues that are very beneficial.

## Introduction

According to the various wide range of reports, the damage caused by the world’s health system—especially to the health of mothers and children—continues to be very serious. To achieve Sustainable Development Goal 3 (SDG 3), that is, ensuring healthy lives and promoting wellbeing at all ages, efforts on the mother’s health during pregnancy, childbirth, and the postpartum period are the main agenda (1). The WHO, and United Nations Children’s’ Fund (UNICEF) advise that women who give birth in medical facilities should have their babies and themselves examined for any health-related challenges within 24 h, considering a date to bring them back for additional postnatal care (PNC) even when everything seems to be going well (2, 3). Furthermore, women should be counseled to return back immediately if they notice any warning signs (4). The initial PNC contact should also be held as soon as practicable, ideally within 24 h of the birth, if the delivery occurs at home. Ultimately, it is advocated that all mothers and newborns receive a minimum of three more postnatal examinations: on day 3 (48–72 h), within days 7–14 immediately after delivery, and 6 weeks later (5–7). Globally, although the number of maternal and infant deaths is decreasing, developing countries, especially, sub–Saharan African (SSA) countries, have a significant share in the failure of the sustainable development goal to reduce 70 maternal deaths per 100,000 live births and 12 neonatal deaths per 1,000 live births by 2030 (8, 9). Since the year 2017, pieces of evidence show that globally, 66% of maternal deaths occur in SSA only (10–12), and the issue is more massive and awful. Inaccessibility to healthcare and inadequate use of postnatal care (PNC) contribute to 99% of maternal mortality in low- and middle-income countries (LMICs), including SSA. These issues are particularly concerning because the majority of the deliveries in these regions occur at home, leading to tragic outcomes that affect public health significantly (9, 12, 13). In comparison to the 230 fatalities per 100,000 live births that occurred in LMICs, the maternal mortality ratio similarly remained unacceptably high at 433 (14, 15). All these gaps could be eliminated by integrating maternal child health service care at a high range (16). Therefore, if women can receive the early postnatal care check-up suggested by the WHO, it could significantly reduce the risk of complications and maternal mortality (6, 17). The SSA countries can avoid greater maternal morbidity and related mortality, which could also help the countries achieve SDG 3.

There are significant gaps in understanding early PNC in East Africa. Key issues include outdated knowledge, lack of region-specific studies, insufficient detail on influencing factors, limited use of advanced statistical methods, and large sample sizes. Addressing these gaps with current, localized, and methodologically robust research is crucial for improving PNC outcomes in the region. Although studies were not conducted under large study settings, numerous studies have found links between the continuum of care and factors such as socioeconomic status, family and individual characteristics, community features, characteristics of the newborn, and interactions with the healthcare system (18, 19). For instance, the age of the mother (20, 21), her marital status (22), her education level (20, 21, 23), occupation (23), the order in which her children were born (20), her access to media (18, 21), residence, health insurance, family number, her ability to make decisions, and her wealth index (19, 21, 24) were found to be the determinants of the continuum of maternal and child care.

Therefore, this study aimed to ascertain the number of reproductive-age women who received each of the chosen functions of the PNC during the first 2 days after birth, as well as its predictors of the number of last live births in the 2 years prior to the survey by applying more advanced and flexible modeling approach to the data. As the Bayesian multilevel approach incorporates prior information, allows for more model flexibility, and is a natural approach to expressing uncertainty, this study benefits the East African countries by identifying key factors—both individual and community-level factors—that influence postnatal care use in the region. It is crucial to enhance PNC and lower rates of child morbidity and mortality. Although studies have not been performed to clarify the elements that support or obstruct PNC at the national level, East African countries continue to be one of the leading causes of mother and infant mortality worldwide (25). The results of this study will provide preliminary information for the upcoming researchers in the area, and policymakers and other stakeholders working on issues related to both women’s and children’s health with an up-to-date multicounty result from which they can build, enhance, and implement their plans.

## Materials and methods

### Study setting and period

For a cross-sectional study design conducted nationwide, data were gathered in East African countries for the period from 2015 to 2022. The study encompassed communities from 10 countries: Burundi, Ethiopia, Kenya, Madagascar, Malawi, Rwanda, Uganda, Tanzania, Zambia, and Zimbabwe. The demographic and health surveys (DHS) Program, funded by the United States Agency for International Development (USAID), provided both financial support and technical assistance for population and health surveys conducted globally. The most recent DHSs dataset encompassing the last 7-year period (2015–2022) in East African nations served as this study’s primary source of information. A standardized dataset was employed (26) to collect a sizable sample size that is representative of the population source and all factors. DHSs gather comparable data on a global scale. The surveys have huge sample sizes, are population-based, and are nationally representative of each nation (26). The 14 nations that make up Eastern Africa are spread throughout the Horn of Africa, the Indian Ocean islands, and the Great Lakes region. These nations struggle with comparable economic, social, and environmental problems and worry that they would not achieve all of the sustainable development objectives (27) East Africa refers to the region of the African continent situated in the Horn and Eastern regions of the Sahara Desert. It is estimated that approximately 486,766,759 individuals inhabit this region, and it spans an area of 6,667,493 km^2^ (2,574,332 sq. mi), constituting around 6.03% of the global population.

### Sample size determination and sampling methods

In every country, the DHSs followed the stratified multistage sampling method, which involves stratification, clustering, and sample selection over two stages. Urban and rural areas were grouped into separate clusters to form the basis for stratification. The administered questionnaire was responded to by women who consented and agreed to participate in the study. Enumeration areas (EAs) inside each sampling stratum were initially chosen with a probability proportional to their size. The households were carefully sampled in the second step. Each of the chosen clusters underwent a household listing operation (28). Demographic and health survey reports were accessible for approximately 10 out of the 13 East African nations. Periodically, every 5 years, a systematic gathering of DHSs surveys is carried out in low- and middle-income countries, utilizing pretested, validated, and structured questionnaires. Using a standardized approach for sampling, questionnaires, data collection, and coding across DHSs surveys enables the possibility of conducting multicountry analyses. In each of the surveys conducted in the specified nations, the most recent conventional census frame was utilized.

The DHSs’ samples are often divided into urban and rural areas within each administrative geographic region. The DHSs employ a stratified two-stage cluster sampling technique. In the first stage, clusters and enumeration areas (EAs) are randomly selected from the sample frame, typically derived from the most recent national census available. Subsequently, systematic sampling is utilized in the second stage to select households within each cluster or EA. During the initial sampling round, EAs are chosen with a probability proportional to the size of each stratum. A predetermined number of households is systematically selected within designated EAs in the second stage. Following the listing of households, equal probability systematic sampling is employed to select a specific number of households within the defined cluster (26).

### Data source and study population

For this study, we utilized DHSs that were conducted over the span of the last 7 years, from 2015 to 2022. Among the approximately 14 countries in East Africa that carried out DHSs during this period, only approximately 10 countries’ surveys contained the necessary information on the outcome variable. Thus, they were included in our analysis. After appending the data from each country, the final analysis included a total weighted sample of 78,213 women and a total unweighted sample of 78,536 women (refer to Table 1). The source population consisted of mothers who had given birth 2 years prior to the survey. Therefore, only mothers who met this criterion were included in the analysis. To account for any unequal sample distributions during the data collection process, weightings were applied to the children’s samples during the estimation process. This adjustment ensured that the results were representative and accurately reflected the studied population.

**Table 1:** List of countries, survey years, and sample size distribution for PNC uptake in East African countries from 2015–2022.

Countries	Survey year	Sample size distributions		
		Unweighted	weighted	Weighted percentage
Burundi	2016/17	8,660	8,821	11.28
Ethiopia	2016	7,193	7,132	9.12
Kenya	2022	5,460	5,486	7.01
Madagascar	2021	9,315	9,176	11.73
Malawi	2015/16	13,448	13,079	16.72
Rwanda	2019/20	6,167	6,313	8.07
Tanzania	2022	5,825	5,963	7.62
Uganda	2016	10,263	9,973	12.76
Zambia	2018	7,372	7,509	9.6
Zimbabwe	2015	4,833	4,761	6.09
Total sample size		78,536	78,213	100

### Data quality control

To ensure data quality in each country, the DHSs implemented several measures. These included providing comprehensive training to data collectors, supervisors, and field editors, as well as conducting ongoing supervision to maintain high standards. Standardized questionnaires were used, which were translated into national and local languages to accommodate participants’ preferred languages. Data processing specialists were involved in the data entry and management phase, ensuring accurate and efficient handling of the collected data. Systematic biases were carefully addressed during this phase to minimize their impact on the results. Once the data were obtained from the DHSs, proper data management procedures were followed. This involved appending women’s and men’s data, addressing missing observations through appropriate techniques such as missing completely at random, and conducting necessary recoding and variable recategorization. These rigorous data management practices were consistently implemented across all countries with similar working systems, ensuring a standardized and reliable approach to data handling and analysis. The DHSs guidance provides additional details regarding the data-collection process. Details can be accessed from the Guide to DHSs statistics (26).

### Variables of the study

#### Dependent variable

The study’s outcome variable is early postnatal care (PNC) within the first 2 days of delivery. To calculate the desired measure of PNC for newborns within the first 2 days after birth, along with the associated variables, the under-five datasets (KR files) from DHSs data of 10 East African countries were utilized as the data source for this analysis. The factors considered for computation included cord examination (m78a), temperature measurement (m78b), counseling on danger symptoms (m78c), breastfeeding counseling (m78d), and nursing observation (m78e) (29). All of these were summed up, and if a newborn did not receive a postnatal check within 2 days of birth, it was recorded as “No (0).” Conversely, if a newborn did receive a postnatal check within 2 days, it was recorded as “Yes (1)“.

#### Independent variables

Both individual and community-level factors that have been identified by earlier researchers and scientific backgrounds as potential factors of PNC checkups were included in this study (Table 2).

**Table 2:** Shows independent variable categorizations among women in East Africa countries, 2015–2022.

Individual level variables	
Variable	Description
Age of the mother	15–24, 25–34, 35–49
Maternal education	no education, primary, secondary and above
Mother working status	Not working, working
Marital status	unmarried, married, Others
Wealth index	Poorest, poorer, middle, richer, richest
Mass media exposure (either watching to television, listening to radio or reading to newspapers/magazines)	No, Yes
Total children ever born	1–2, 3–5, 6 and above
Place of delivery	Home, Health facility
At least one ANC visit	No, Yes
Women perception to distance to the health facility	Not big problem, big problem
Weight at birth	Small, average, and large
Sex of a child	Male, Female
Twin	Single, Multiple
Birth order	1st, 2nd to 3rd, ≥4th
Community level variables	
Residence	Urban, Rural
Community women education	Low, High
Community wealth level	Low, High
Community media exposure	Low, High
Community ANC utilization	Low, High
Community level distance to health facility	Not big problem, big problem

#### Data management and analysis process

We conducted a secondary study using the Kids Records (KR) dataset, which involved analyzing the data from the DHSs. To carry out the analysis, we utilized R 4.3.2 software (68, 69) and Microsoft Excel version 19 for tasks such as data downloading, appending, cleaning, recoding, and handling missing values. In the DHSs’ data, infants were nested within clusters, and neonates within the same cluster showed more remarkable similarity than those in different clusters. This violated the assumptions of independence of observations and equal variance across clusters in the classical logistic regression model. Therefore, a more sophisticated modeling approach was required to account for between-cluster factors. We constructed a Bayesian multilevel random logistic regression model to investigate the relationship between individual- and community-level variables and the risk of not receiving immediate PNC within 2 days of birth. In total, four models were developed. The first model, known as the empty or null model, was fitted without any explanatory variables. This model helped reduce community differences and served as a baseline for understanding community variances. It also assisted in determining whether a multilevel statistical framework should be used instead of a standard logistic regression model. The null model was evaluated using various measures, including the log–likelihood ratio (LLR), median odds ratio (MOR), intraclass correlation coefficient (ICC), proportional change of variance (PCV), and the widely applicable information criterion (WAIC). The second model included only individual or household-level characteristics, while the third included only community-level factors without individual-level factors. Finally, the fourth and final model included components from both the individual and community levels. These models were constructed to assess the impact of individual and community factors on mothers’ behavior regarding immediate PNC. Then, we conducted a Bayesian meta-analysis to compute the pooled prevalence and construct the forest plot (30). Subsequently, a two-level Bayesian logistic regression was employed to identify the determinants of PNC.

#### Bayesian mixed model and convergence of algorithm

To identify the factors influencing early PNC uptake, we utilized multilevel mixed-effects logistic regression analysis employing the Bayesian approach. The Bayesian approach to credible intervals serves a similar purpose to classical confidence intervals (CIs); however, their composition and interpretation philosophies differ significantly (31). The Bayesian statistical approach offers the ability to incorporate additional prior information external to the data through prior distributions. By leveraging this additional prior information, the accuracy and credibility of effect size estimations can be improved. Consequently, applying the classical confidence interval interpretation is inappropriate, as the Bayesian statistical approach provides a more reasonable alternative. The Bayesian credible interval interpretation is more intuitive in this context, while frequentist confidence intervals are often misinterpreted as Bayesian credible intervals.

We used the random variable EAs to account for the variation of early postnatal care (PNC) utilization across different EAs within the country. An ICC value >5% was used as a threshold to consider the variation across EAs. Since the outcome variable was dichotomized (no/yes), children within households were treated as level-1 unit, while EAs were considered as level-2 units. This hierarchical structure resulted in children being nested within EAs. We employed a Bayesian multilevel logistic regression model to address the hierarchical nature of the data and account for the dependency of observations within the same cluster. This approach allowed us to obtain accurate and credible estimates of effect sizes, considering the clustering of children within EAs and the potential impact of the EA on the outcome variable.

Hence, the dependent variable was represented by:


$$Y_{ij}=\{\begin{array}{l} 1,\mathrm{if}\;\mathrm{the}\;ith\;\mathrm{women}\;\mathrm{in}\;\mathrm{the}\;jth\;EA\;\mathrm{received}\;\mathrm{early}\;PNC\\ 0,\mathrm{if}\;\mathrm{the}\;ith\;\mathrm{women}\;\mathrm{in}\;\mathrm{the}\;jth\;EA\;\mathrm{have}\;\mathrm{not}\;\mathrm{received}\;\mathrm{early}\;PNC \end{array}$$

*The likelihood function*

The key ingredients to a Bayesian analysis are the likelihood function, which reflects information about the parameters contained in the data, and the prior distribution, which quantifies what is known about the parameters before observing data. The prior distribution and likelihood can be easily combined to form the posterior distribution, which represents total knowledge about the parameters after the data have been observed. Bayesian multilevel logistic analysis specifies a dichotomous dependent variable as a function of a set of explanatory variables (32, 33).

Likelihood contribution from the ith subject in the jth group is Bernoulli:


$$L\left(\theta_{i}\right)=\theta_{i}^{y_{i}}{\left(1-\theta_{i}\right)}^{1-y_{i}}\text{,}$$

where $\theta_{i}$ represents the probability of the event for subject i in group j that has a covariate vector $x_{i}$ and $y_{i}$ indicates the presence ( $y_{i}=1$ ) or absence ( $y_{i}=0$ ) of the event for that subject. In multilevel logistic regression, we know that


$$\theta_{i}=\mathrm{logi}t^{-1}\left(\beta_{0}+\beta_{1}x_{i1}+\beta_{2}x_{i2}+\ldots +u_{0j}+\gamma_{0}+\gamma_{1}x_{i1}+\gamma_{2}x_{i2}+\ldots +v_{0j}\right)\text{,}$$

where $\beta_{0}+\beta_{1}x_{i1}+\beta_{2}x_{i2}+\ldots +\beta_{k}\;X_{ijk}$ is a fixed part of the model and $u_{0j}$ is a random part of the model and $u_{0j}∼N\left(0,\sigma_{u}^{2}\right)$ . $\theta_{i}$ is the probability of the ith child in the jth the group having PNC uptake so that the likelihood contribution to the $i^{th}$ subject in the jth group is


$$\begin{array}{l} L\left(\theta_{i}|\;,x_{i}|\;,\sigma_{u}^{2}\right)={\left(\frac{\exp\left(\begin{array}{l} \beta_{0}+\beta_{1}x_{i1}+\beta_{2}x_{i2}+\ldots \\ +u_{0j}+\gamma_{0}+\gamma_{1}x_{i1}+\gamma_{2}x_{i2}+\ldots +v_{0j} \end{array}\right)}{1+\exp\left(\begin{array}{l} \beta_{0}+\beta_{1}x_{i1}+\beta_{2}x_{i2}+\ldots \\ +u_{0j}+\gamma_{0}+\gamma_{1}x_{i1}+\gamma_{2}x_{i2}+\ldots +v_{0j} \end{array}\right)}\right)}^{y_{i}}\cdot \\ {\left(1-\frac{\exp\left(\begin{array}{l} \beta_{0}+\beta_{1}x_{i1}+\beta_{2}x_{i2}+\ldots +u_{0j}\\ +\gamma_{0}+\gamma_{1}x_{i1}+\gamma_{2}x_{i2}+\ldots +v_{0j} \end{array}\right)}{1+\exp\left(\begin{array}{l} \beta_{0}+\beta_{1}x_{i1}+\beta_{2}x_{i2}+\ldots +u_{0j}\\ +\gamma_{0}+\gamma_{1}x_{i1}+\gamma_{2}x_{i2}+\ldots +v_{0j} \end{array}\right)}\right)}^{1-y_{i}}\text{.} \end{array}$$

Since individual subjects in the group are assumed to be independent of each other, the likelihood function over a dataset of n subjects in the jth group is then


$$L\left(\theta |\;,\;x|\;,\;\sigma_{u}^{2}\right)=\overset{n}{\underset{i=1}{\prod }}\overset{11}{\underset{j=1}{\prod }}\left[\begin{array}{l} {\left(\frac{\exp\left(\begin{array}{l} \beta_{0}+\beta_{1}x_{ij1}+\beta_{2}x_{ij2}+\ldots +u_{0j}+\gamma_{0}\\ +\gamma_{1}x_{ij1}+\gamma_{2}x_{ij2}+\ldots +v_{0j} \end{array}\right)}{1+\exp\left(\begin{array}{l} \beta_{0}+\beta_{1}x_{ij1}+\beta_{2}x_{ij2}+\ldots +u_{0j}+\gamma_{0}\\ +\gamma_{1}x_{ij1}+\gamma_{2}x_{ij2}+\ldots +v_{0j} \end{array}\right)}\right)}^{y_{ij}}\\ \cdot {\left(1-\frac{\exp\left(\begin{array}{l} \beta_{0}+\beta_{1}x_{ij1}+\beta_{2}x_{ij2}+\ldots +u_{0j}\\ +\gamma_{0}+\gamma_{1}x_{ij1}+\gamma_{2}x_{ij2}+\ldots +v_{0j} \end{array}\right)}{1+\exp\left(\begin{array}{l} \beta_{0}+\beta_{1}x_{ij1}+\beta_{2}x_{ij2}+\ldots +u_{0j}\\ +\gamma_{0}+\gamma_{1}x_{ij1}+\gamma_{2}x_{ij2}+\ldots +v_{0j} \end{array}\right)}\right)}^{1-y_{ij}} \end{array}\right]$$

*Prior distribution*

For a flat normal prior, we will assume a normal distribution for the coefficients $\beta_{k}$ and $U_{0j}$ with mean 0 and variance $\sigma^{2}$ .

Let us denote the prior distribution for the parameters as follows:


$$P\left(\beta_{k}\right)∼\mathrm{Normal}\left(0,\sigma^{2}\right)$$

$$P\left(U_{0j}\right)∼\mathrm{Half Cauchy}\;\left(0,\sigma^{2}\right)$$

*Posterior distribution*

The full conditional distribution for parameter $\beta_{0}$ is given by (34):


$$P\left(\beta_{k}|\;,\delta_{u0}^{2}|\;,Y_{ij}\right)\propto \underset{j}{\prod }\theta_{ij}^{Y_{ij}}{\left(1-\pi_{ij}\right)}^{1-Y_{ij}}\propto \underset{j}{\prod }\left[{\left(\frac{e^{\beta_{k}+U_{0j}}}{1+e^{\beta_{k}+U_{0j}}}\right)}^{Y_{ij}}{\left(\frac{1}{1+e^{\beta_{0}+U_{0j}}}\right)}^{1-Y_{ij}}\right]$$

For parameter $\delta_{uo}^{2}$ , the full conditional distribution is (34):


$$\begin{array}{l} P\left(\delta_{uo}^{2}|\;,\beta_{k}|\;,Y_{ij}\right)\propto \underset{j}{\prod }{\left(\pi_{ij}\right)}^{Y_{ij}}{\left(1-\pi_{ij}\right)}^{1-Y_{ij}}\\ \times \;\mathrm{inverse}\;\mathrm{gamma}\;\left(n/2+n\left(\alpha -1\right)\;,n\beta \right) \end{array}$$

$$\begin{array}{l} P\left(\delta_{uo}^{2}|\;,\beta_{k}|\;,Y_{ij}\right)\propto \underset{j}{\prod }{\left(\pi_{ij}\right)}^{Y_{ij}}{\left(1-\pi_{ij}\right)}^{1-Y_{ij}}\\ \times \left(\frac{\beta^{\alpha }}{\Gamma \left(\alpha \right)}\right)\times x^{-\alpha -1}e^{-\beta /x}I\left(x>0\right) \end{array}$$

where n is the total number of observations.

To estimate the parameters of the variable and the extent of random variations between clusters, we used the Brms-R package (70–72). It uses Hamiltonian Monte-Carlo (HMC) and its extensions No-U-Turn Sampler (NUTS) that uses a recursive algorithm to build a set of likely candidate points that spans a wide swath of the target distribution, stopping automatically when it starts to double back and retrace its steps. These features allow it to converge to high-dimensional target distributions much more quickly than simpler methods, such as the random walk Metropolis or Gibbs sampler (35). Currently, in a multilevel framework, Brms provides an intuitive, powerful, and flexible formula syntax that extends the well-known formula syntax of lme4, especially when modeling categorical and ordinal data (36). Hence, we employed this package to fit multilevel logistic regression models based on the Bayesian approach.

The regression coefficients for each parameter and variance were estimated using a flat prior with a normal distribution (0, 1,000) and a half-Cauchy distribution (0, 25), respectively. In addition, we employed iteration = 4,000, warm-up (number of discarded iterations) = 2,000, cores = 2 (specifying the number of cores used for the algorithm), chains = 2, adapt delta (controlling divergent transitions) = 0.95, and initials (starting values for the iterations) = 0 to estimate the posterior distribution.

For the multilevel binary logistic regression analysis, four models were developed. These models included an empty model, which had no independent variables and served to assess the extent of cluster variation in early PNC utilization. Additionally, there were models with individual-level variables, community-level variables, and a full model incorporating both types of variables. The widely applicable information criterion (WAIC) was utilized to identify the best-fitting model,. This criterion is preferred over the commonly used deviance information criterion (DIC) for model selection (37, 38). A model with the lowest WAIC was the best fitted model. The results obtained from a given HMC analysis are not believed to be reliable until the chain has reached its stationary distribution and converged much more quickly (39, 40). Hence, to assess the convergence diagnostics criteria, we employed the following indicators: (37, 73, 74) Rhat = 1, effective sample sizes (both Bulk_ESS and Tail_ESS) exceeding 1,000. Additionally, we examined the time series plots to ensure well-mixed chains, observed smooth density plots, and verified that the autocorrelation plots showed minimal correlation with zero for the majority of parameters (Supplementary Figures S1–S3).

### Measure of variations between levels

To estimate the EA effects on early PNC uptake outcome and to quantify the variation in the early PNC uptake outcome between EAs (i.e., clusters), we applied the variance partition coefficient (VPC) and the median odds ratio (MOR), respectively.

*Intra-class correlation (ICC)*: The VPC measures the proportion of the total observed individual variation in the outcome that is explained by the between cluster variation. The VPC requires an estimate of the variance at the individual level (level 1), and for the standard logistic distribution, this variance is equal to $\frac{\pi^{2}}{3}$ (41). Therefore, in the multilevel logistic regression models M1 and M2 with a logit link, the PCV or ICC is approximated by $ICC=\frac{\sigma^{2}\mu }{\sigma^{2}\mu +\frac{\pi^{2}}{3\text{.}}}$

where *σ*^2^*μ* is the variance of the random parameter at the cluster level representing the amount of unobserved heterogeneity between clusters; and $\frac{\pi^{2}}{3}$ is a parameter representing the amount of unobserved heterogeneity between women (individual-level variance).

*Median Odds Ratio (MRR)*: The MOR measures how much variability in the early PNC uptake exists between EAs (i.e., clusters) by comparing two persons from two randomly chosen, different EAs (42). Consider two persons chosen randomly from two different EAs but with the same values of covariates in the model. The MOR is the median odds ratio between the person at EA with a higher probability of receiving early PNC services and the person at Survey EA with a lower probability of receiving early PNC services. It is a function of the estimated EAs, that is, cluster, variance $\sigma^{2}\mu$ and is given by


$$\text{MOR}=\exp\;{(}^{\sqrt{2\sigma^{2}}}\times f^{-1}\left(\frac{3}{4}\right)),$$

where *σ*^2^ is the variance of each model, and *ƒ*^−1^ is the inverse of the standard normal cumulative distribution function ${ƒ}^{-1}\;\left(0.75\right)$ is the 75th percentile, and $\exp\left(\text{.}\right)$ is the exponential function (42, 43).

*Proportional change in variance* (*PCV*) was calculated as $PCV=\frac{\left(V_{0}-V_{x}\right)\times 100}{V_{0}}$ , where *V*_0_ is the variance of the null model, and *V_x_* is the variance of each model at each level with variables (41).

### Operational definitions for community-level factors

#### Community women education

This metric reflects the collective value of women’s educational attainment, which is determined based on the median distribution of educational levels within the neighborhood. If the proportion of women in the community with at least a secondary education fell below the median, it was considered low, while if it exceeded the median, it was considered high. On average, the median proportion was 0.50.

#### Community media exposure

This variable was created based on individual responses regarding exposure to radio or television media. If the proportion of women in the community who reported media exposure was between 0 and 48%, it was classified as low. Conversely, if the proportion ranged from 49 to 100%, it was considered high.

#### Community ANC utilization rate

This variable is derived from individual data on antenatal care (ANC) utilization. If the proportion of women in the community who attended at least one ANC visit fell between 0 and 50%, it was classified as low. The median value for this proportion was 0.51.

#### Community perception distance to health facility

This community-level variable was constructed based on individual responses regarding the major challenge in accessing health facilities, specifically related to the distance to the nearest facility. If the proportion of women in the community who perceived distance as a significant problem to visit health facilities ranged from 0 to 14%, it was categorized as a low problem. The median value for this proportion was 0.15.

#### Community wealth

The procedure employed to obtain this variable was similar to that used for the household’s wealth index. If the proportion of women in a community belonging to the two lowest wealth quintiles ranged from 50 to 100%, it was classified as high. Conversely, if the proportion fell between 0 and 49%, it was considered low.

## Results

### Sociodemographic characteristics of the study participants

In this analysis, a total of 78,213 weighted numbers of mothers were included. Out of the total mothers interviewed 44.7% of them were between 25 and 34 years of age. Close to 49% of the women had primary education, while 29% had secondary and above education. The majority (64%) of mothers were employed, and 67% were married. In addition, 45.67% of the participants were poor, and about 57% had media exposure. Nearly 93% of the women had ANC visits during their pregnancy time. Moreover, 77% of them gave birth at a health facility. About 58% of mothers give birth for the first time at the age of 19 years and below. Only 20% of the households had a number of under-5-year-old children above the age of 5 years. Close to half (51%) of the children were males, and only 1.7% were twins. Approximately 53.47% of the children had average size, and 41% of the children were the fourth or above child in the family. Regarding community-level variables, nearly 53% of the participants were from four countries (namely, Burundi, Madagascar, Malawi, and Uganda), and 77% were rural residents. Approximately 53% of the population had low exposure to mass media. Nearly 51% of women in the community had low educational levels. Regarding the community utilization of health facility delivery was low at 52%. Approximately 52% of the community had low ANC utilization and poverty level, respectively (Table 3).

**Table 3:** Participant characteristics early PNC uptake and its associated factors following child birth in East Africa: using recent DHS data from 2015 to 2022.

Variables	Frequency (weighted)	Percentage (weighted)
Maternal age		
15–24	24,662	31.53
25–34	34,960	44.7
35–49	18,590	23.77
Maternal education		
Not primary education	16,954	21.68
Primary education	38,488	49.21
secondary and Higher	22,771	29.11
Marital status		
Unmarried	5,441	6.96
Married	52,648	67.31
Others*	20,124	25.73
Wealth index		
Poorest	19,318	24.7
Poorer	15,637	19.99
Middle	14,470	18.5
Richer	14,228	18.19
Richest	14,560	18.62
Media exposure		
No	44,545	56.95
Yes	33,668	43.05
Age at first birth		
< 19 years	45,072	57.63
≥20 years	33,141	42.37
Mother working		
No	28,071	35.89
Yes	50,142	64.11
Number of under five children		
1–2	33,494	42.82
3–5	30,106	38.49
above 5	14,613	18.68
At least one ANC visit		
No	5,524	7.06
Yes	72,689	92.94
Place of delivery		
Home	18,111	23.16
Health facility	60,102	76.84
Twin		
No	76,885	98.3
Yes	1,327	1.7
Weight at birth		
Large	23,261	29.74
Average	41,819	53.47
Small	13,133	16.79
Sex of the child		
Male	39,719	50.78
Female	38,494	49.22
Distance to health facility		
Not big problem	30,239	38.66
Big problem	47,974	61.34
Birth order		
1st	17,741	22.68
2nd or 3rd	28,682	36.67
4th and above	31,790	40.65
Residence		
Urban	18,088	23.13
Rural	60,125	76.87
Community ANC utilization		
Low	40,288	51.51
High	37,925	48.49
Community educational level		
low	39,903	51.02
High	38,310	48.98
Community distance to health facility		
Not long	71,405	91.3
Long	6,808	8.7
Community media exposure		
low	41,286	52.79
High	36,927	47.21
Community wealth level		
Low	39,516	50.52
High	38,697	49.48

### Pooled magnitude of early PNC uptake in East Africa

**Figure 1:**
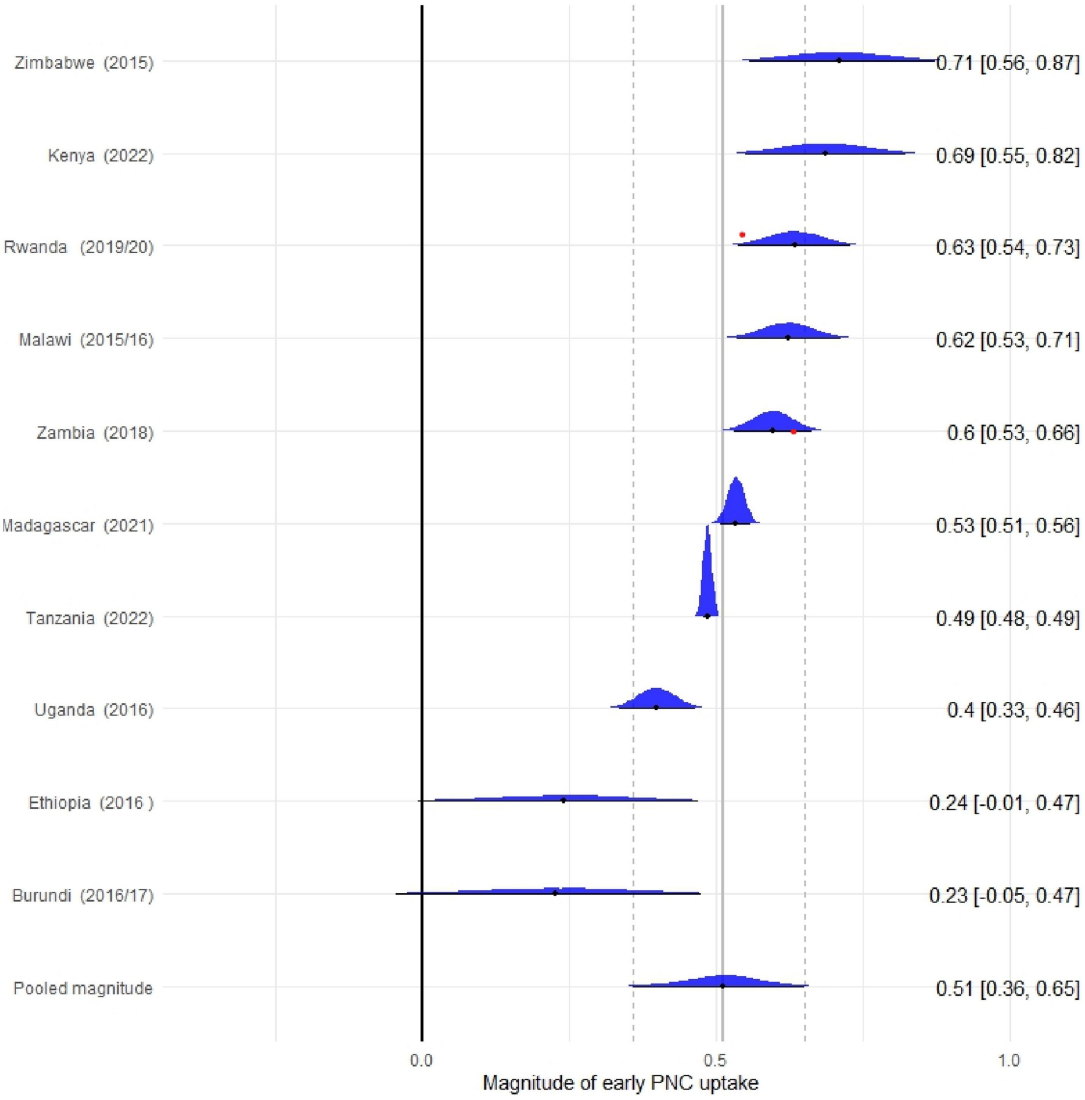
Forest plot of pooled prevalence of early PNC uptake in East Africa across 10 countries from 2015 to 2022.

The pooled magnitude of early PNC uptake in the East African countries was 52% (95% CI: 39, 66). The highest magnitude of early PNC uptake was found in Zimbabwe (71%), followed by Kenya (69%) and Rwanda (63%). The lowest magnitude of early PNC utilization was reported in Uganda (40%). The between-country difference in magnitude of early PNC uptake (*τ*) was 22% (95% CI: 11, 44) (Figure 1).

### Measures of variation (random effect estimates) analysis

The model with both individual and community-level factors has the smallest Widely Applicable Information Criteria (WAIC = 98,050.2) compared to the random intercept-only model (WAIC 107,081.7), the model with only individual-level factors (WAIC = 98,361.9), and the model with only community-level factors (WAIC = 105,612). Therefore, this model is the best fitted model for the data because it has the smallest WAIC compared to the other models. Interpretations and reports were made based on this model. The random effect estimates have been determined by fitting four models (Model I (null model), Model II, Model III, and Model IV). The null model showed significant variability in the likelihood of early PNC uptake among East African countries (*σ*^2^ = 0.202). The ICC of 0.06 in the null model implied that the difference between clusters contributed 6% of the total variation in the uptake of early PNC services. The MOR of 1.54 in the null model implied that if children were picked randomly from two different clusters (EAs), children within a cluster had a higher probability of receiving early PNC, with a 1.54 times higher chance of receiving early PNC services compared to children within a cluster with a lower chance of uptake of early PNC services. The proportional change in variance (PCV) in the full model was 55.7%, showing that both individual- and community-level factors explained 55.7% of the observed variation in uptake of early PNC (see Table 4).

**Table 4:** Random effect analysis of PNC among women in East Africa.

Model comparisons parameters	Model I	Model II	Model III	Model IV
Cluster level variance	0.2	0.09	0.14	0.09
ICC	0.06	0.03	0.04	0.03
PCV	Ref	55.65%	31.18%	55.7%
MOR	1.54	1.33	1.43	1.34
WAIC	107,081.7	98,361.9	105,612	98,050.2

### Factor affecting early PNC uptake

In a multilevel multivariable Bayesian logistic regression analysis, variables, namely, maternal age, maternal education, marital status, mother’s working status, wealth index, media exposure, age at first birth, parity, place of delivery, at least one ANC, birth size, birth order number, while residence, community media exposure, community wealth level, community ANC utilization and community distance to health facility were identified as significant individual and community-level factors of receiving early PNC service (Table 5).

**Table 5:** Individual and community level factors associated with early PNC uptake among women in East African countries using the recent DHS datasets, 2015–2022.

Early PNC uptake	Estimate	Est. Error	AOR (95% CrI)	Rhat	Bulk_ESS	Tail_ESS
Maternal age						
15–24	Ref					
25–34	0.19	0.02	1.21 (1.15, 1.27)*	1	3,200	3,353
35–49	0.47	0.03	1.61 (1.5, 1.72) *	1	3,003	2,886
Maternal education						
Not primary education	Ref					
Primary education	0.67	0.02	1.96 (1.88, 2.05) *	1	4,248	3,453
secondary and Higher	1.16	0.03	3.19 (3.03, 3.36) *	1	4,343	3,082
Marital status						
Unmarried	Ref					
Married	−0.04	0.03	0.97 (0.9, 1.03)	1	3,783	3,041
Others **	−0.19	0.03	0.83 (0.77, 0.89)*	1	3,727	3,206
Mother working status						
Not working	Ref					
Working	−0.1	0.02	0.9 (0.87, 0.93)*	1	7,903	2,810
Wealth index						
Poorest	Ref					
Poorer	−0.13	0.02	0.88 (0.84, 0.92)*	1	7,039	2,722
Middle	−0.19	0.02	0.83 (0.79, 0.87)*	1	3,686	2,948
Richer	−0.26	0.03	0.77 (0.73,0.81)*	1	3,211	3,289
Richest	−0.53	0.03	0.59 (0.55, 0.63)*	1	3,195	2,669
Media exposure						
No	Ref					
Yes	0.28	0.02	1.32 (1.27, 1.36)*	1	7,039	2,722
Parity						
1–2	Ref					
3–5	−0.12	0.03	0.89 (0.84, 0.94) *	1	2,910	2,761
Above 5	−0.36	0.04	0.69 (0.64, 0.75) *	1	3,143	3,209
Age at first birth						
< 19 years	−0.2	0.02	0.82 (0.79,0.84) *	1	3,784	3,217
≥20 years	Ref					
ANC visit						
No	Ref					
Yes	0.66	0.04	1.93 (1.8, 2.08) *	1	6,705	3,115
Place of delivery						
Home	Ref					
Health facility	0.94	0.02	2.57 (2.46, 2.68) *	1	6,673	3,155
Child sex						
Male	Ref					
Female	0.01	0.02	1.01 (0.98, 1.04)	1	7,187	2,774
Weight at birth						
Large	Ref					
Average	−0.06	0.02	0.94 (0.91, 0.98) *	1	6,339	3,212
Small	−0.13	0.02	0.88 (0.84, 0.92) *	1	5,814	3,394
Twin						
No	Ref					
Yes	0.14	0.06	1.15 (1.02, 1.3) *	1	8,324	2,810
Birth order						
1st	Ref					
2nd or 3rd	−0.01	0.03	0.99 (0.95, 1.05)	1	3,622	2,894
4th and above	−0.12	0.04	0.88 (0.82, 0.95) *	1	2,959	2,791
Residence						
Urban	Ref					
Rural	−0.28	0.02	0.76 (0.72, 0.79)*	1	5,292	3,014
Community educational level						
low	Ref					
High	0.01	0.03	1.01 (0.95, 1.08)	1	2023	2,697
Community media exposure						
low	Ref					
High	0.1	0.03	1.1 (1.04, 1.18) *	1	1924	2,644
Community ANC utilization						
Low	Ref					
High	0.12	0.03	1.13 (1.07,1.19) *	1	2,297	3,018
Community wealth						
Low	Ref					
High	−0.13	0.03	0.88 (0.83, 0.94) *	1	2039	2,801
Community distance to health facility						
Big problem	Ref					
Not big problem	0.41	0.04	1.5 (1.38, 1.63) *	1	2,524	2,699

NB: AOR, Adjusted odds ratio; CrI, Credible interval; ESS, Effective sample size, *statistically significant variable, and **divorced/widowed.

The odds of early PNC service uptake among women in the age group of 25–34 years (AOR = 1.21; 95% CrI: 1.15, 1.27) and 35–49 years (AOR = 1.61; 95% CrI: 1.5, 1.72) were higher by 21 and 61%, respectively, compared to women aged 15–24 years old. Similarly, women who had achieved primary education (AOR = 1.96; 95% CrI: 1.88, 2.05) and secondary/higher education (AOR = 3.19; 95% CrI: 3.03, 3.36) were 1.96 and 3.19 times more likely to receive early PNC services than formally uneducated women, respectively. Divorced or widowed women (AOR = 0.83; 95% CrI: 0.77, 0.89) had 17% lower odds of early PNC service uptake than unmarried women. Currently working women (AOR = 0.9; 95% CrI: 0.87, 0.93) had 10% lower odds of early PNC service uptake than those not working. Regarding household wealth indexes, taking the poorest households as a reference, poorer women (AOR = 0.88; 95% CrI: 0.84, 0.92), middle-class women (AOR = 0.83; 95% CrI: 0.79, 0.87), richer (AOR = 0.77; 95% CrI: 0.73, 0.81), and richest women (AOR = 0.59; 95% CrI: 0.55, 0.63) all had decreased odds of early PNC service uptake as wealth increased. Women with media exposure (AOR = 1.32; 95% CrI: 1.27, 1.36) had 32% higher odds of early PNC service uptake than those without media exposure. Women with 3–5 children (AOR = 0.89; 95% CrI: 0.84, 0.94) and those with more than 5 children (AOR = 0.69; 95% CrI: 0.64, 0.75) had lower odds of early PNC service uptake compared to those with one to two children. Women with an age at first birth <20 years (AOR = 0.82; 95% CrI: 0.79, 0.84) had slightly lower odds of early PNC service uptake compared to those with an age at first birth ≥20 years. Women with at least one ANC visit (AOR = 1.93, 95% CrI: 1.8, 2.08) had 93% higher odds of early PNC service uptake than those without an ANC visit. Women delivering in health facilities (AOR = 2.57; 95% CrI: 2.46, 2.68) were 2.57 times more likely to receive early PNC service uptake than those at home. Women with a child who had an average birth size (AOR = 0.94; 95% CrI: 0.91, 0.98) and small birth size (AOR = 0.88; 95% CrI: 0.84, 0.92) had slightly lower odds of early PNC service uptake compared to those with large birth size. Women with twins (AOR = 1.15; 95% CrI: 1.02, 1.3) had 15% higher odds of early PNC service uptake than those without twins. Fourth- or above-born children (AOR = 0.88; 95% CrI: 0.82, 0.95) had slightly lower odds of early PNC service uptake compared to first-born children. Regarding community-level variables, rural women (AOR = 0.76; 95% CrI: 0.72, 0.79) had lower odds of early PNC service uptake by 24% compared to urban women. Communities with high media exposure (AOR = 1.1; 95% CrI: 1.04, 1.18) had 10% higher odds of early PNC service uptake than their counterparts. Communities with high wealth levels (AOR = 0.88; 95% CrI: 0.83, 0.94) had 12% lower odds of early PNC service uptake. Communities with high ANC utilization (AOR = 1.13; 95% CrI: 1.07, 1.19) had 13% higher odds of early PNC service uptake than those with low ANC utilization. Communities in which the distance to a health facility is not a big problem (AOR = 1.5; 95% CrI: 1.38, 1.63) had 50% higher odds of early PNC service uptake than communities where it is a big problem (Table 5).

## Discussion

Using the Bayesian multilevel modeling approach, the goal of this study was to pinpoint the underlying individual and community-level factors that contributed to the PNC uptake among mothers immediately following birth within 24–48 h. Based on our final model analysis, we found that the pooled prevalence of PNC following birth within 2 days of the delivery was about 52% (95% CrI: 39, 66). Although this pooled prevalence was still low, however, it has been shown to have approximately 20% increase as compared to the previous research subgroup analysis findings in SSA (44). In comparison to the prevalence recorded in Ethiopia (15.71%) (45), and Gambia (46), this prevalence was greater. Although the prevalence of PNC has increased over time in East Africa, this number is not very spectacular when compared to other countries such as Bangladesh (63%) (47), Benin (68%) (48), and Zambia (63%) (49). These differences may reflect the variety of country-specific factors, analysis model type, sampling, and other methodological changes, as well as the effectiveness of the health care system delivering mother and child care.

In this study, educated and older mothers have more PNC than their counterparts. According to a study conducted in SSA, Vietnam, and China, educated and older mothers have more PNC than their counterparts (44, 50, 51). Several previous studies have shown that a mother’s education and age have a significant impact on a child’s health (52). It is closely linked to her knowledge of health, which influences her use of maternal and child health services. Women with higher levels of education and age tend to have better knowledge about maternal health, enabling them to make informed decisions and seek appropriate medical care, including maternal health services. Higher education and being aged are associated with improved health-seeking behaviors, being more susceptible to maternal- and pregnancy-related complications, and are seen as a crucial way to empower women to access maternal healthcare services (53, 54). Therefore, the evidence suggests that educated and older women are more likely to have better knowledge and utilize maternal health services because they understand the importance of these services and can make informed choices.

When enabling factors are taken into account, mothers who had at least one ANC visit and gave birth in health facilities had a higher chance of getting a PNC checkup than their counterparts, which is supported by earlier research from India (55), Pakistan (56), Ethiopia (45, 57), Bangladesh (47) as well as Nigeria (58), and Uganda (59). This finding could be explained by the fact that pregnant women who receive counseling and information about the health risks of the postpartum period during ANC visits are more likely to use adequate PNC services, which maintains their advantageous position regardless of their socioeconomic status. This finding might be explained by the fact that pregnant women who are exposed to counseling and information about the health risks of the postpartum period during ANC visits and during their delivery at medical facilities are more likely to use adequate PNC services, which maintain their advantageous position regardless of their socioeconomic status. Additionally, these women who gave birth in a hospital might be allowed to stay for an additional 2 days or advised to have a follow-up appointment 2 days after giving birth so that they can receive the proper medical attention for both themselves and their newborns. Mothers who give birth at home typically face cultural restrictions that prevent them from leaving their homes for a certain amount of time, exhibit negative attitudes, and use fewer postpartum services.

Although previous research did not provide specific explanations for why widowed mothers show less health-seeking behavior for themselves and their children, it can be inferred from the broader literature that the loss of a spouse has a significant impact on a woman’s motivation and ability to seek healthcare. This impact includes increased levels of depression, anxiety, and psychological distress, which can affect a woman’s willingness to access healthcare for herself and her children (60). Additionally, widowed mothers often lack social support, making it more difficult for them to seek healthcare (60). Moreover, the death of a spouse can lead to economic hardships, which further hinder a woman’s ability to access healthcare for herself and her children. Furthermore, in some communities, widows may face stigmatization or discrimination, which can negatively influence their healthcare-seeking behavior (61). While the specific reasons for the lower health-seeking behavior among widowed mothers may vary depending on individual circumstances and cultural contexts, it is crucial to offer targeted support and interventions to address the unique challenges faced by this population.

The majority of the studies have investigated those urban women who had more PNC than rural women due to the unequaled distribution of sources and various factors of women. Several factors could explain this difference. One reason is that rural areas often have limited transportation options and poor road infrastructure (62). This makes it difficult for women to travel to healthcare facilities for PNC visits. Additionally, rural women may have less information about PNC services and their importance, which can lead to lower utilization rates (62). Rural women may also face more socioeconomic challenges, such as poverty and a lack of resources, which can prevent them from seeking PNC services. Furthermore, rural areas typically have fewer healthcare facilities and resources, resulting in longer travel and wait times for PNC services, and women may face challenges in attending PNC visits due to the distance of the nearest health facility from their homes (63). Finally, in some rural communities, there may be cultural or social norms that discourage women from seeking PNC services (64). For example, there might be a preference for traditional birth attendants or a belief that PNC visits are unnecessary. All these could be caused by differences in the accessibility and availability of healthcare facilities, as well as the caliber of healthcare provided in the various regions.

This study showed that mothers who come from areas with high levels of media exposure and who frequently consume any form of media, including radio and television, are more likely to use PNC from any provider, regardless of qualification, than their counterparts who are not exposed to any media. This outcome is consistent with the research performed in Bangladesh (47), Uganda (59), Ethiopia (65), and Nigeria (66), where only qualified providers were considered. The fact that highly educated women who are already aware of the seeking behavior are more likely to be exposed to media can be used to explain this better occurrence. This demonstrates how the country can improve communication hurdles, PNC use, and other health services by leveraging the power of media.

Compared to those at parity 1, women at parity 4 and more had reduced probabilities of using PNC for their offspring. The most likely explanation is that women with higher parities may rely on the knowledge they gained from prior PNC visits. Studies from Uganda and Bangladesh have shown their agreement with this figure (47, 59). On the contrary, it is believed that a variety of obligations, such as raising younger and older children, frequently prevent women from seeking PNC, particularly in situations where access to a health facility is regarded to be difficult, mainly if they have more children and have not used contraception.

Compared to women who had single infants, twin mothers were more likely to seek PNC. Utilizing PNC services is crucial since they provide health professionals with a platform to identify and treat problems affecting expectant mothers and newborns. Women must use these services as a result, regardless of the manner of birth. Perhaps the stigma associated with twin pregnancies pushes women to seek PNC; however, more research on twin pregnancies and PNC uptake is required to fully understand the phenomena. Currently, studies from Bangladesh and Papua New Guinea have discovered a comparable number (47, 67). In the same way, if their babies are overweight or have obesity-like tendencies that are different from the babies they knew before and around them, they do not consider it as a good opportunity, so they are more likely to go to the health facility for a checkup.

This study found that wealthier and employed mother have a less likelihood chance of having PNC within 2 days following birth compared to their counterparts. According to a study conducted in Vietnam and SSA, women in wealthier households were significantly associated with postnatal health checks for their newborns. The proportion of postnatal health checks increased among households of near-poor, middle, rich, and richest as compared with the poorest households (44, 50). Although the author did not find earlier evidence, several factors could explain why wealthier and employed mothers may not return to health facilities within 2 days after giving birth, compared to poor and unemployed women. These factors include access to underreported private healthcare, self or relative postpartum support, dissatisfaction, and cultural or personal preferences. Wealthier women may have other options for postpartum care, such as home visits from healthcare providers. Cultural or personal preferences may also play a role in when women choose to visit health facilities after giving birth. Additionally, employed mothers may face challenges in taking time off work for these visits. These factors contribute to the disparities in postpartum health facility visits among different socioeconomic groups. Further research and data analysis on this topic would provide a more comprehensive understanding of the underlying reasons.

### Strengths and limitations of the study

Regarding the study’s advantages, East African countries’ nationally representative data, which is indicative of the entire nation, is used. To offer a more believable conclusion that considers the hierarchical nature of the survey data by incorporating prior information, this study also used a Bayesian multilevel modeling technique and sizable samples. This will improve the estimation of the results toward the reality of the country’s current status. The study does have some flaws, though. Due to the self-reported nature of the interview, the survey is subject to social desirability, and the cross-sectional form of the study may not be able to account for the temporal connection of the independent and outcome variables. Furthermore, although there was a relatively short time variation among the countries in the dataset, time was not considered an independent factor in the regression. Therefore, readers should keep this in mind when interpreting and using the findings of this study.

### Conclusion, recommendations, and implications of the study

Low utilization of immediate PNC uptake remains among the top public health importance agendas in East Africa. In the final model, the study at hand revealed several crucial individual and community-level factors, including older maternal age, higher education levels, being divorced/widowed women, better household wealth index, maternal working status, having mass media exposure, higher parity, having ANC follow up, institutional delivery, age at first birth, twin status, child weight at birth, and birth order were individual-level factors that have been independently associated with early PNC uptake. Similarly, regarding community-level factors, variables such as high community mass media exposure, being from a rural area, being from high ANC follow-up coverage communities, being from high-wealth communities and having a long distance to the health facilities were associated with the outcome variable, respectively, among women in East Africa.

Hence, targeted education and awareness campaigns are crucial. Given the positive correlation between higher education levels and early PNC uptake, it is important to implement campaigns that specifically target women with lower levels of education. These campaigns should emphasize the benefits of PNC and highlight the importance of seeking PNC services early in the postnatal period. Additionally, they should address any misconceptions or barriers that may exist. Similarly, it is important to strengthen ANC and institutional delivery services. The study has identified ANC follow-up and institutional delivery as factors that are positively associated with early PNC uptake. By improving access to quality ANC services, ensuring the presence of skilled birth attendants, and establishing adequate referral systems, the likelihood of women receiving timely PNC can be increased. Furthermore, community-based interventions are also crucial. The study has found that community-level factors, such as mass media exposure and ANC follow-up coverage, can influence early PNC uptake. Therefore, implementing community health education programs and utilizing mass media platforms to disseminate PNC-related information can help improve awareness and promote early PNC-seeking behaviors. The future research should explore how the identified factors influence early PNC uptake and evaluate the effectiveness of interventions targeting these factors. Longitudinal studies could provide insights into the long-term impact of early PNC uptake on maternal and child health outcomes. Overall, the study underscores the importance of addressing both individual and community factors to enhance early PNC uptake in East Africa. By implementing targeted interventions and policies, healthcare systems can ensure that all women have equal access to timely and high-quality PNC services, improving maternal and child health outcomes.

## Data Availability

The raw data supporting the conclusions of this article will be made available by the authors, without undue reservation.
