# Supplementary material for: Early postnatal care uptake and its associated factors following childbirth in East Africa—a Bayesian hierarchical modeling approach
Source: Front Public Health. 2024 Nov 27;12:1439280. doi: 10.3389/fpubh.2024.1439280 (PMC11632800; doi:10.3389/fpubh.2024.1439280)
Supplement: Supplementary file 1 [file Supplementary_file_1.docx]

**Supplementary files**


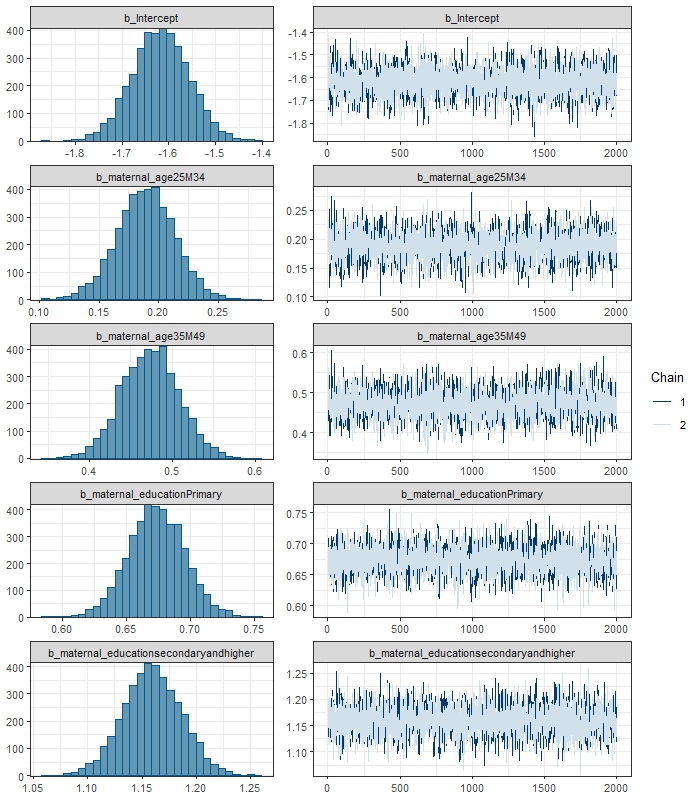


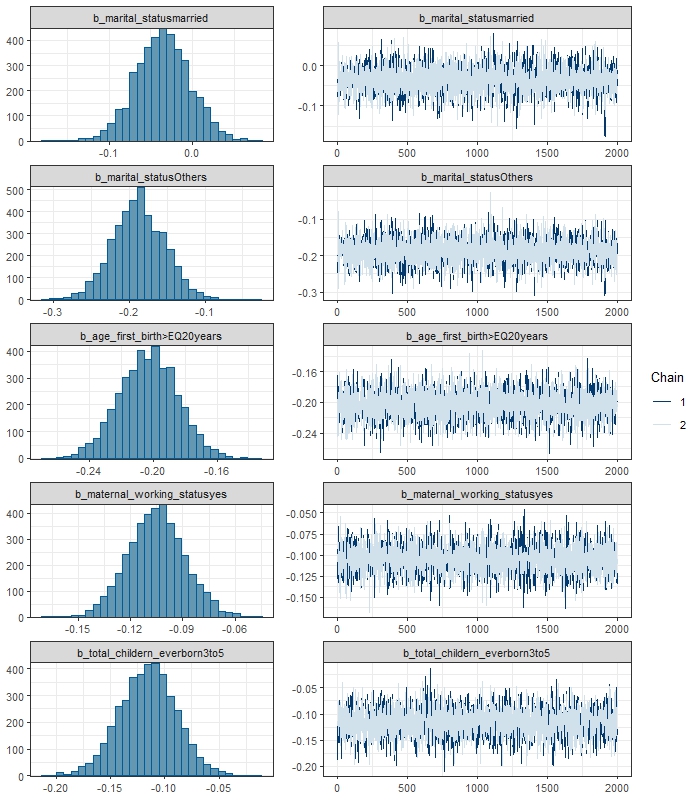


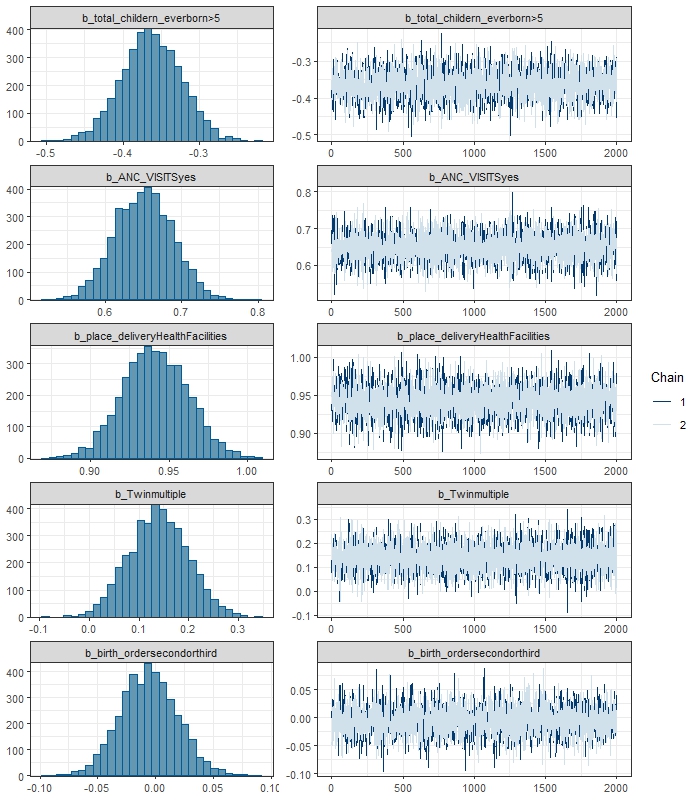


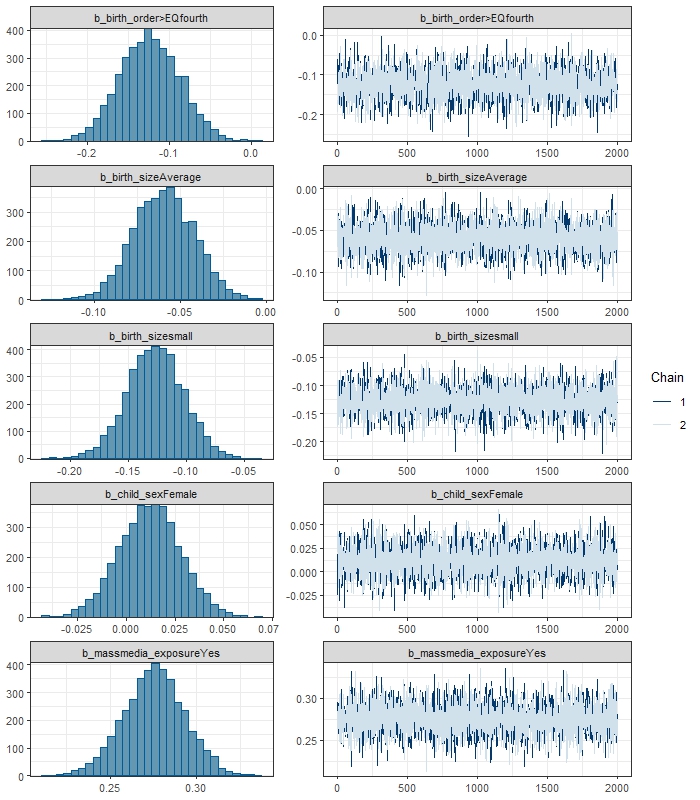


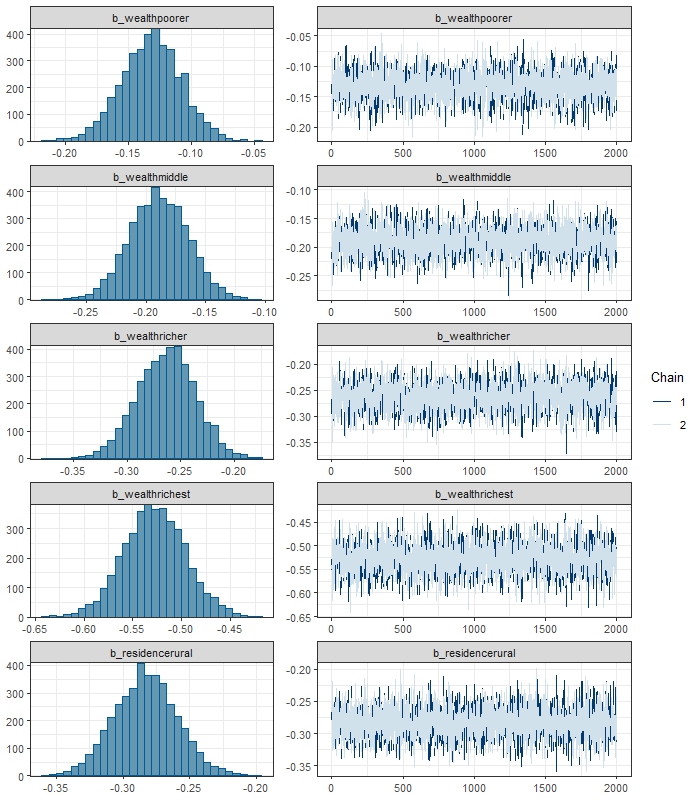


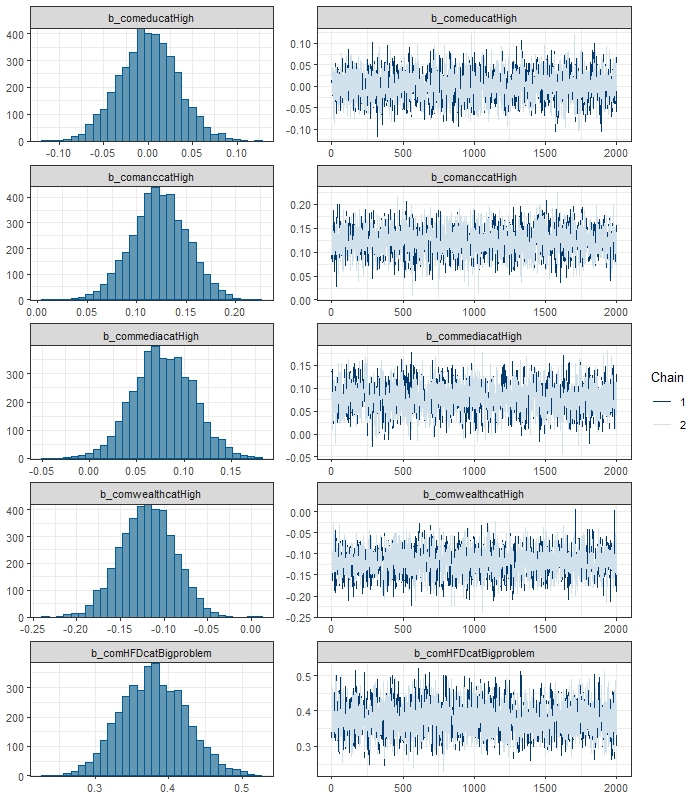


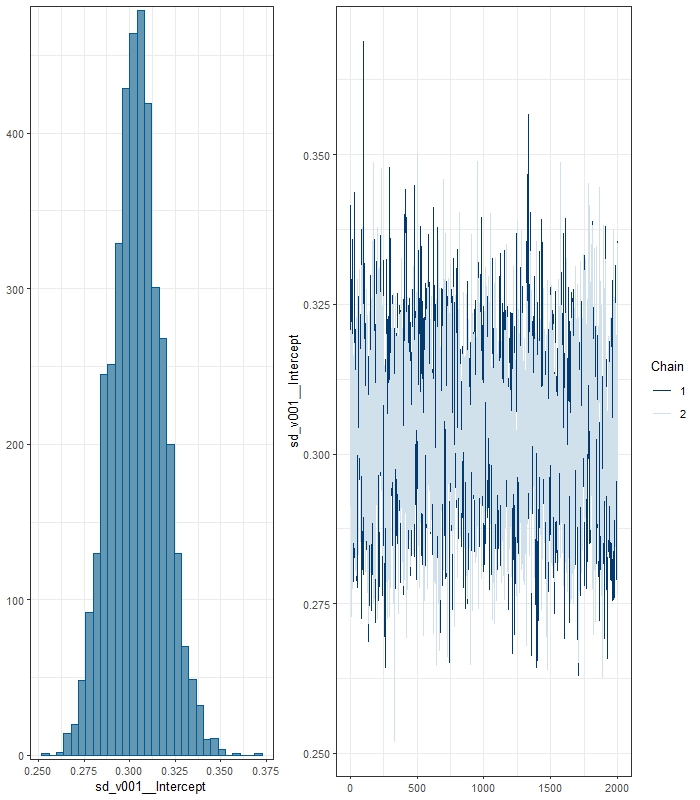


fig S1: Histogram and time series trace plots for all the paraments in the final model of early PNC uptake in East Africa using recent datasets, 2015-2022.


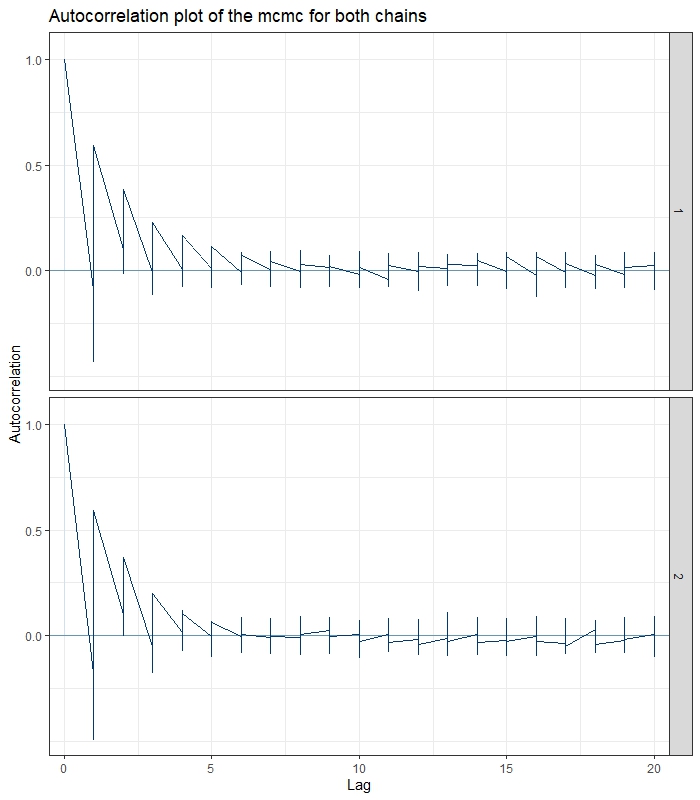


fig S2: Autocorrelation function plot for the chains in the MCMC process for the final model of early PNC uptake in East Africa using recent datasets, 2015-2022


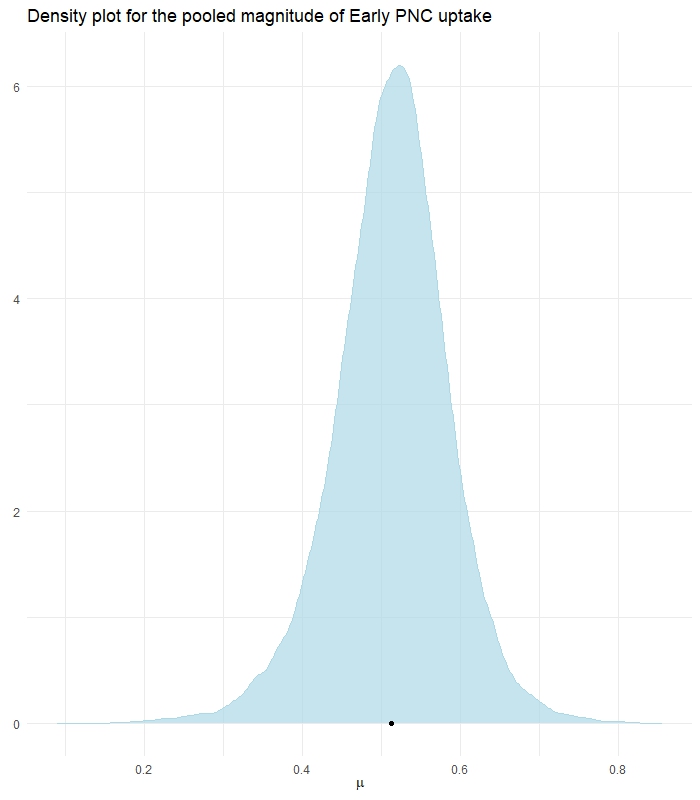


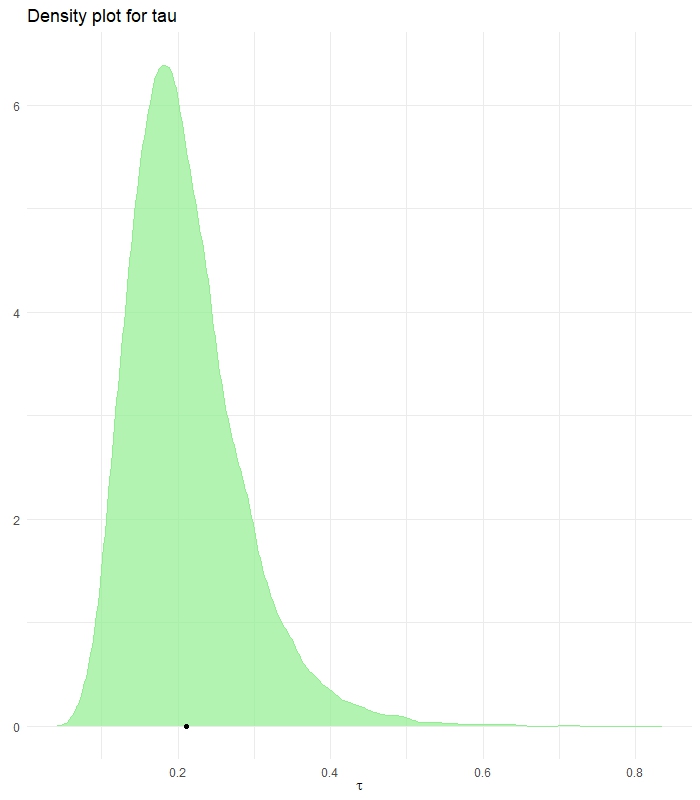


fig S3: The Density plot for the pooled magnitude $\mu$ and variance ($\tau)$of early PNC uptake in East Africa using recent datasets, 2015-2022
